# A Flexible Side-Chain Dispersant Enables Uniform Self-Assembled Monolayers for 18.67% Organic Solar Cells

**DOI:** 10.3390/molecules31132321

**Published:** 2026-07-02

**Authors:** Mengmeng Wang, Shibo Wang, Yabing Tang, Yuyan Li, Mengyu Qiu, Heng Liu, Leying Zha, Yajing Zhang, Xinhui Lu, Guilong Cai

**Affiliations:** 1 School of Chemical and Molecular Sciences, Henan University (Zhengzhou), Zhengzhou 450000, China; 2 Longzihu New Energy Laboratory, Zhengzhou 450000, China; 3 Department of Physics, The Chinese University of Hong Kong, New Territories, Hong Kong 999077, China; 4 Beijing Key Laboratory of Solid State Battery and Energy Storage Process, Institute of Process Engineering, Chinese Academy of Sciences, Beijing 100190, China

**Keywords:** self-assembled monolayers, molecular dispersant, aggregation suppression, interfacial modification

## Abstract

Self-assembled monolayers (SAMs) on indium tin oxide (ITO) surfaces tend to undergo molecular aggregation, resulting in non-uniform interfacial coverage and thus limiting the charge extraction efficiency and device performance in organic solar cells (OSCs). To address this issue, a novel dispersant molecule, 3,6-Diiodo-9-[2-(2-methoxyethoxy) ethyl]-9H-carbazole (2ICzMPE), featuring dipolar characteristics and flexible side chains, is designed and synthesized to regulate the interfacial distribution of 2PACz molecules on ITO electrodes. With a large dipole moment and steric hindrance, 2ICzMPE suppresses 2PACz aggregation, while the oxygen atoms in the side chains interact with the ITO surface, enabling a more uniform interfacial structure. Upon introducing 2ICzMPE, the interfacial energy level alignment is optimized, leading to more efficient charge extraction. Device physics analysis reveals suppressed carrier recombination and enhanced charge transport. As a result, the power conversion efficiency (PCE) of OSCs based on the PM6:L8-BO system is improved from 17.51% to 18.67%. This work provides a simple and effective molecular design strategy for constructing high-quality SAM interfaces and promoting the scalable application of OSCs.

## 1. Introduction

Organic solar cells (OSCs) have attracted extensive attention as promising next-generation photovoltaic technologies owing to their light weight, mechanical flexibility, tunable optical transparency, and compatibility with low-cost solution processing. In recent years, the power conversion efficiency (PCE) of OSCs has achieved remarkable improvements, wherein interface engineering plays a key role in further optimizing charge transport and suppressing non-radiative recombination [1,2,3]. Among the various functional layers, the hole transport layer (HTL) plays a critical role in regulating interfacial energy level alignment, facilitating hole extraction, and influencing the morphology and electronic properties of the active layer. Poly(3,4-ethylenedioxythiophene):poly(styrenesulfonate) (PEDOT:PSS) has long been the most widely used HTL in OSCs owing to its excellent film-forming ability and favorable conductivity. However, with the continuous improvement of OSC performance, the intrinsic limitations of PEDOT:PSS have become increasingly evident. The parasitic optical absorption of this material decreases photon absorption, and the limited nature of its energy-level tuning also limits its ability to be compatible with new donor–acceptor systems. Even more seriously, PEDOT:PSS is acidic and hygroscopic, which can degrade the indium tin oxide electrode (ITO) and thus threaten the stability of the device. These disadvantages have prompted the quest for alternative HTL materials that have better interfacial behavior [4,5]. In order to alleviate these shortcomings, self-assembled monolayers (SAMs) have become extremely attractive as HTL materials due to their minimal parasitic absorption and tunable energy level [6]. However, SAM molecules usually have low adsorption capacity and high intermolecular forces, which tend to lead to molecule aggregation and non-uniform interfacial coverage [7,8]. Consequently, it remains challenging to form uniform, compact, and highly ordered monolayers on ITO surfaces. Such interfacial inhomogeneity can induce charge accumulation and exacerbate recombination, particularly in large-area devices. Therefore, despite the excellent efficiencies achieved with SAM-based OSCs at the laboratory scale, their photovoltaic performance typically deteriorates significantly upon device scaling, presenting a major hurdle for the commercialization of organic photovoltaics [9].

In order to overcome such problems, different approaches have been designed to control SAM assembly, which can be broadly divided into the molecular design and interfacial engineering methods. On the molecular level, anti-aggregation design is commonly implemented to inhibit excess intermolecular interactions and facilitate orderly packing. For example, Du et al. have added sterically hindered SAM molecules to decrease the strength of the π–π stacking, allowing the creation of dense and well-ordered monolayers and also minimizing non-radiative recombination losses [10]. At the interfacial level, co-adsorption strategies are widely used to optimize SAM assembly. Similarly, Li et al. incorporated a co-adsorbent molecule (PyCA-3F) into the 2PACz system, which effectively suppresses aggregation and improves interfacial uniformity [11]. Bin et al. regulated the length of the carazole SAM alkyl chain with a phosphonic acid anchor group and obtained 2/3/4PACz as the hole transmission layer. Compared with PEDOT:PSS, the efficiency of light transmission, resistance, and the device is better. 3PACz has the best comprehensive performance, adapts to a variety of receptors, and has good application prospects [12]. Chou et al. proposed an interspersed assembled monolayer (IAM) strategy employing dipolar dispersants (NNN and NSN) with structures analogous to the host SAM. Through push–pull interactions and optimized dipole moments, this approach suppresses micelle formation, improves energy-level alignment, facilitates hole extraction, and reduces recombination, thereby enhancing the efficiency and stability [13]. Another alternative strategy is multicomponent interfacial assembly. As an example, Ma et al. added covalent organic frameworks (COFs) at the interface and several intermolecular interactions were combined to enhance the interfacial uniformity and charge transport [14]. Solvent engineering has also been applied to control the solution state aggregation in the process of film formation. Particularly, the formation of micelles of SAM molecules was prevented by modifying solvent polarity using co-solvent systems, leading to smooth and more dense interfacial layers [15]. Nevertheless, even with these developments, there are still numerous schemes that have difficulty with their complexity, cost, and limited scalability. Therefore, developing simple, low-cost, and scalable interfacial modifiers with broad applicability remains highly desirable.

In this work, we designed and readily synthesized a novel dispersant molecule, 2ICzMPE, via a simple one-step reaction. The molecule features dipolar characteristics and methoxyethoxy side chains and serves to regulate the interfacial assembly behavior of SAMs. The addition of 2ICzMPE is effective in eliminating molecular aggregation by means of intermolecular push–pull and increased steric hindrance. In addition, it enhances the density of hydroxyl-related active sites on the surface of ITO, stabilizes oxygen vacancy defects, and enhances interfacial anchoring. With 2ICzMPE, we obtain a uniform SAM featuring an optimized Fermi level. This interfacial structure favors hole extraction, suppresses charge recombination, and reduces energy losses. Benefiting from these synergistic effects, OSCs (PM6:L8-BO) incorporating 2ICzMPE as an interfacial regulator exhibit a substantially improved PCE increasing from 17.51% to 18.67%. This work presents a simple, low-cost, and scalable molecular engineering strategy to overcome the long-standing challenge of SAM-related interfacial inhomogeneity in organic photovoltaics. This method offers a promising pathway to the scalable interfacial engineering of high-performance OSCs and has great prospects in application.

## 2. Results and Discussion

### 2.1. Molecular Design and Interfacial Regulation

**Figure 1:**
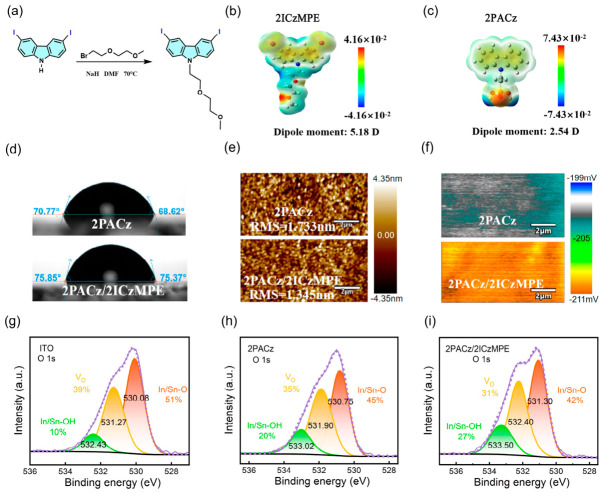
(**a**) Molecular structure of 2ICzMPE and schematic illustration of SAM regulation; (**b**,**c**) electrostatic potential (ESP) distribution of the 2ICzMPE molecule; (**d**) water contact angle measurements of ITO modified with 2PACz and 2PACz/2ICzMPE; (**e**) AFM height images of ITO surfaces modified with 2PACz and 2PACz/2ICzMPE; (**f**) surface potential (KPFM) maps of ITO modified with 2PACz and 2PACz/2ICzMPE; (**g**–**i**) XPS spectra of pure ITO surface and XPS spectra of ITO surfaces modified with 2PACz and 2PACz/2ICzMPE.

Conventional SAMs tend to aggregate on the ITO surface, leading to non-uniform coverage and reduced interfacial stability. Previous studies have shown that SAM molecules can form micelles or aggregates during solution processing due to hydrophobic interactions and π–π stacking, which hinder their ordered assembly on electrode surfaces [16]. To solve this problem, a novel interspersed assembled monolayer, 2ICzMPE, was designed and synthesized. It can be synthesized in one step, and the raw materials are inexpensive, as depicted in Figure 1a. The structure of the 2ICzMPE was confirmed via NMR and HRMS spectroscopy (Figures S1–S3). The thermogravimetric analysis (TGA) was performed to evaluate the thermostability of 2ICzMPE, and the TGA curves are shown in Figure S4. During device fabrication, 2ICzMPE was introduced at a concentration ratio of 1:10 relative to 2PACz to regulate SAM assembly behavior. The molecule adopts a carbazole core, providing good conjugation and compatibility with SAM molecules, while methoxyethoxy side chains are introduced at the nitrogen atom of the carbazole unit [9]. The larger dipole moment of 2ICzMPE, which is structurally similar to 2PACz, enables push–pull interactions that decrease micelle formation. At the same time, dispersants equipped with extended side chains provide greater steric hindrance, thereby facilitating 2ICzMPE dispersion and strengthening their ability to disrupt the aggregation of 2PACz micelles [17]. In addition, the oxygen atoms in the side chains possess lone pairs that might coordinate with oxygen vacancies on the ITO surface, which may in turn contribute to the increased anchoring of 2PACz to the substrate and potentially improved adsorption stability [18]. This interaction not only facilitates the formation of a uniform SAM but also passivates surface defects and modulates the local electronic distribution, thereby optimizing the Fermi level of ITO and improves energy level alignment [19].

Electrostatic surface potential (ESP) maps from the density functional theory (DFT) calculations at the B3LYP/6-31G(d,p) level reveal a pronounced asymmetric charge distribution for 2ICzMPE (Figure 1b,c), with positive potential primarily localized on the oxygen atoms and negative potential distributed over the carbazole core, indicating a strong intrinsic molecular dipole that creates a robust interfacial dipole to modulate energy-level alignment and facilitate charge extraction [20]. Notably, 2ICzMPE possesses a substantially higher dipole moment of 5.18 D than that of 2PACz (2.54 D), reflecting its considerably stronger polarity. Such a large molecular dipole is advantageous for establishing a more favorable interfacial electric field, thereby facilitating interfacial charge transport and contributing to improve device performance [21].

In addition, the effect of 2ICzMPE on the SAM interface was further investigated through water contact angle measurements (Figure 1d). The water contact angle increases from 8.63° for bare ITO (Figure S5) to 69.70° after 2PACz deposition and further to 75.61° upon introducing 2ICzMPE, indicating enhanced hydrophobicity [10]. This change is likely related to improved SAM coverage and an optimized molecular orientation. Specifically, 2ICzMPE suppresses the self-aggregation of 2PACz, leading to the formation of a more uniform molecular layer on the ITO surface. Additionally, it may further demonstrate that the molecular packing becomes more ordered, with hydrophobic carbazole moieties oriented toward the air interface and hydrophilic phosphonic acid groups anchored onto the ITO surface [8]. Given that the introduction of 2ICzMPE modulates the assembly behavior and interfacial distribution of SAM molecules, we characterized the film surface using atomic force microscopy (AFM) [22]. The AFM results (Figure 1e) show that the root-mean-square (RMS) (Figure S6) roughness decreases from 1.733 nm to 1.345 nm upon introducing 2ICzMPE, indicating a smoother and more uniform interface [23]. Consistently, surface potential mapping via Kelvin probe force microscopy (KPFM, Figure 1f) reveals a more homogeneous potential distribution after introducing 2ICzMPE. Moreover, the average surface potential shifts slightly from −201 mV to −211 mV. This phenomenon is attributed to more uniform SAM packing upon 2ICzMPE incorporation, which together contribute to a more uniform interfacial electronic landscape. Such a change is expected to facilitate energy-level alignment and enhance charge extraction [24,25].

Based on the differences in interfacial assembly behavior, X-ray photoelectron spectroscopy (XPS) was performed on samples with different interface modifications to analyze the evolution of their chemical state (Figure 1g), wherein a comprehensive quantitative analysis of hydroxyl oxygen (In/Sn-OH, around 532.4 eV), oxygen vacancy (V_O_, around 531.3 eV), and lattice oxygen (In/Sn-O, around 530.1 eV) was conducted with deconvolution of the O 1s spectra [7,26]. Compared with the treated ITO substrate, the 2PACz-covered substrate exhibits a higher proportion of hydroxyl oxygen (10% vs 20%) (Figure 1h), but a lower proportion of oxygen vacancy (39% vs. 35%). In contrast, the ITO substrate modified with 2ICzMPE exhibits a further increase in hydroxyl oxygen (27%) coupled with a further reduction in oxygen vacancy (31%) (Figure 1i). In addition, compared with bare ITO, the O 1s peaks corresponding to hydroxyl oxygen, oxygen vacancy, and lattice oxygen all show a systematic blueshift toward higher binding energies [27]. Similar binding energy shifts are also observed in the In 3d and P 2p spectra (Figure S7). These observed spectral shifts can be attributed to electrostatic effects arising from the larger dipole moment of 2ICzMPE, which increases the uniformity of 2PACz on the underlying ITO substrate. Similarly, this trend aligns well with the findings from the KPFM analysis presented above [25].

### 2.2. Device Performance and Energy-Level Regulation

**Figure 2:**
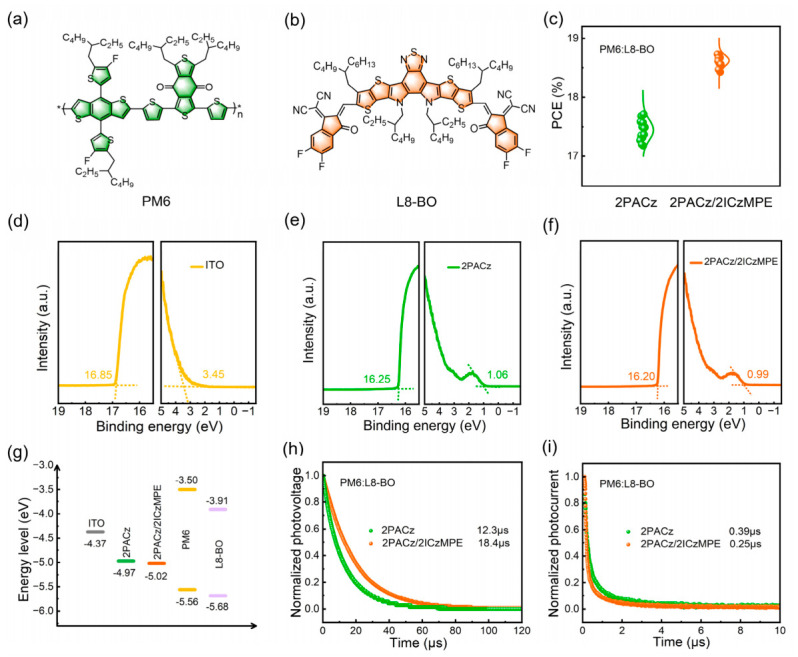
(**a**,**b**) Device architecture of OSCs based on the PM6:L8-BO system; (**c**) *J*-*V* characteristics and photovoltaic performance statistics of devices with different interface modifications; (**d**–**f**) secondary electron cutoff region of UPS spectra; (**g**) valence band region of UPS spectra; (**h**) transient photovoltage (TPV) measurements; (**i**) transient photocurrent (TPC) measurements. The asterisks (*) indicate the polymerization sites that connect adjacent repeating units along the PM6 polymer backbone.

The PM6:L8-BO (Figure 2a,b) system features favorable energy-level alignment and complementary absorption, enabling efficient charge transport and photovoltaic conversion [28,29]. Consequently, it was selected as a model system to investigate the influence of interface engineering on device performance, given its well-established photovoltaic characteristics and broad applicability in the literature. The self-aggregation of 2PACz molecules on the ITO surface leads to non-uniform coverage and local defects in the self-assembled monolayer, which in turn causes interfacial charge accumulation and recombination [30]. Prior to determining the optimal composition, a series of solutions with different molar ratios were systematically investigated (Table S1). As a result, the devices based on the conventional 2PACz interface exhibit an open-circuit voltage (*V*_OC_) of 0.859 V, a *J*_SC_ of 25.88 mA cm^−2^, and FF of 78.77%, yielding a PCE of 17.51% with a broad performance distribution among individual devices, reflecting poor interfacial uniformity (Table S2, Supporting Information). Upon introducing the 2ICzMPE, the PCE of devices increases to 18.66% (*V*_OC_ = 0.867 V, *J*_SC_ = 26.71 mA cm^−2^, FF = 80.62%), while the performance distribution narrows significantly, indicating enhanced reproducibility (Figure 2c). We attribute this improvement to 2ICzMPE effectively suppressing the formation of large micelles, thereby enabling 2PACz to anchor as a valid hole-selective layer on the ITO surface. Moreover, the larger dipole moment of 2ICzMPE further facilitates charge extraction mediated by 2PACz, resulting in an enhanced *J*_SC_ [31]. Correspondingly, the *J*-*V* curve shown in Figure S8a and external quantum efficiency (EQE) spectra (Figure S8b) reveal that both devices exhibit high photoresponse across the entire spectral range, consistent with its higher *J*_SC_ [32]. Similar trends were also observed on other high-performance SAM substrates, where the PCE of Me-4PACz increased from 17.37% to 18.47%, while that of the 4PADCB substrate increased from 17.46% to 18.45% (Table S3, Supporting Information).

To further elucidate the origin of the improved device performance from the perspective of interfacial energetics, we characterized the evolution of the interfacial energy-level structure using ultraviolet photoelectron spectroscopy (UPS) and observed a slight yet systematic shift in the Fermi level (*E*_F_) of the ITO substrates. As shown in Figure 2d–f, the *E*_F_ of ITO exhibits systematic variations under different interface conditions. Compared with the bare ITO (*E*_F_ = −4.37 eV), the *E*_F_ of ITO/2PACz and ITO/2PACz + 2ICzMPE shifted downward to −4.97 and −5.02 eV (Figure 2g), corresponding to an increased work function of the ITO electrode. This is corroborated by KPFM surface potential mapping, which shows a more homogeneous potential distribution and a shift in the average surface potential. The enhanced work function reduces the energy offset between the electrode Fermi level and the active layer HOMO, thereby strengthening the built-in field at the interface. The introduction of the strong dipole molecule 2ICzMPE further reinforces the surface dipole layer [33,34]. The additional molecular dipole aligns cooperatively with the 2PACz dipole, pushing the vacuum level downward. The *E*_F_ of the ITO electrode shifts downward to a more negative value. Notably, it is the relative position of this *E*_F_ with respect to the HOMO of the active layer that primarily governs hole extraction. A lower *E*_F_ reduces the hole-extraction barrier at the electrode/active layer interface, thereby promoting interfacial charge collection and suppressing carrier recombination [35,36,37,38]. Consequently, efficient hole transport is facilitated, energy losses are minimized, and overall device performance is improved.

Transient photovoltage (TPV, Figure 2h) and transient photocurrent (TPC, Figure 2i) together provide direct insight into trap-mediated recombination and the effective carrier lifetimes under device operation. TPV characterizes charge recombination kinetics, where the decay lifetime reflects the carrier lifetime, indicating suppressed charge recombination and a prolonged carrier lifetime. The results show that with 2ICzMPE, the TPV decay lifetime is significantly extended from 12.3 μs to 18.4 μs. The reduced *E*_F_ (from −4.97 eV to −5.02 eV) induced by the strong dipole molecule 2ICzMPE strengthens the built-in electric field at the anode interface. A stronger built-in field facilitates spatial separation of photogenerated carriers and reduces interfacial charge recombination, which is consistent with the observed TPV lifetime increase [39]. Meanwhile, TPC evaluates the charge extraction process, with the decay time indicating the carrier transport and extraction rate, demonstrating faster and more efficient charge extraction [40,41]. The TPC decay time is shortened from 0.39 μs to 0.25 μs. The above results indicate that the regulation of 2PACz distribution by 2ICzMPE not only optimizes the interfacial structure and energy-level alignment but also significantly improves charge carrier transport and suppresses recombination, thereby synergistically enhancing the overall photovoltaic performance of the devices.

### 2.3. Active Layer Morphology

**Figure 3:**
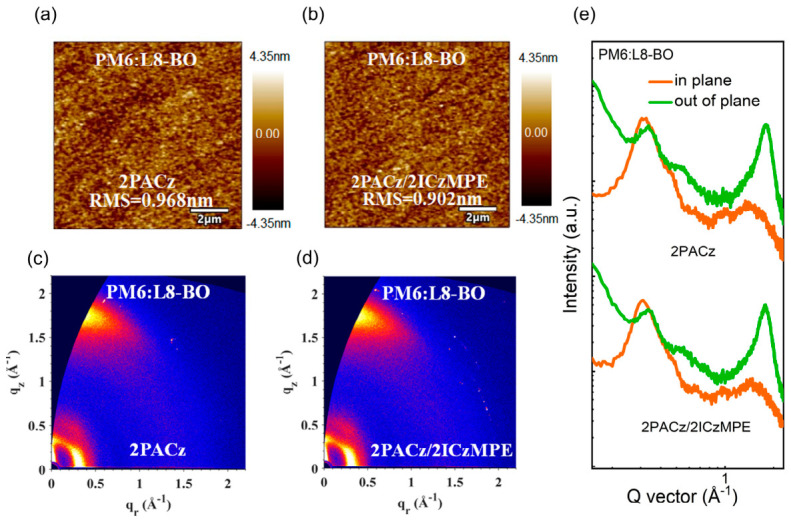
(**a**,**b**) AFM height images of PM6:L8-BO active layers deposited on different SAM-modified ITO substrates; (**c**,**d**) two-dimensional GIWAXS patterns of the active layers; (**e**) corresponding one-dimensional GIWAXS profiles.

In addition to interfacial energy-level regulation, the properties of the SAM interface also affect the morphology and molecular packing of subsequently deposited active layers. The surface morphology and molecular packing of PM6:L8-BO active layers under different interface modification conditions were systematically characterized to reveal the effect of the dispersant on the microstructure of the active layer. As depicted in Figure 3a,b, based on the ITO/2PACz interface, the RMS roughness of the PM6:L8-BO active layer was approximately 0.968 nm. Upon incorporation of the 2ICzMPE into the 2PACz, the more uniform SAM surface enhances the wettability of the active layer, thereby reducing the RMS roughness to 0.902 nm [42]. Further line-scan analysis of the AFM images revealed that the average fibril width decreased from 133.8 nm to 129.4 nm (Figure S9) [43,44]. These results indicate that the interface-engineered SAM provides a more uniform environment for the active layer, leading to improved film quality.

As shown in Figure 3c–e, we used Grazing-Incidence Wide-Angle X-ray Scattering (GIWAXS) to examine potential structural changes in the active layer caused by SAM modification. Compared with 2PACz, the π–π stacking peak shows no shift after introducing 2ICzMPE, remaining at approximately 1.77 Å^−1^. Correspondingly, the d-spacing is about 3.55 Å (Table S4, Supporting Information) [45,46]. Meanwhile, the full width at half maximum (FWHM) decreased from 0.330 to 0.285, and the coherence length (CL) increased from 1.71 nm to 1.98 nm. The decreased FWHM and increased coherence length indicate a slight improvement in the molecular ordering and crystallinity of the active layer after introducing 2ICzMPE, which is beneficial for charge transport, thereby contributing to the overall device performance improvement [47,48,49,50].

### 2.4. Device Physics Analysis

**Figure 4:**
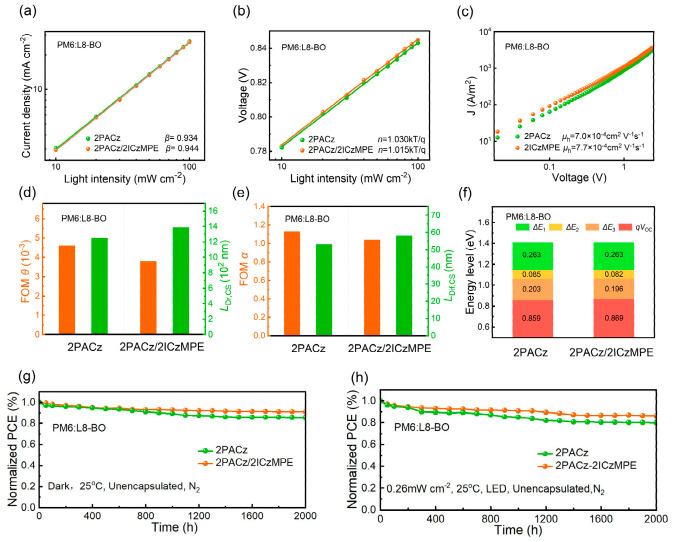
(**a**) Relationship between short-circuit current density (*J*_SC_) and light intensity for devices; (**b**) relationship between open-circuit voltage (*V*_OC_) and light intensity for devices; (**c**) hole-only device structure and space-charge-limited current (SCLC) characteristics for mobility evaluation; (**d**,**e**) schematic illustration and quantitative analysis of charge transport lengths under drift and diffusion processes; (**f**) energy loss decomposition and statistical analysis of photovoltaic parameters; (**g**,**h**) storage stability and light stability of the unencapsulated devices.

In addition, we investigated the dependence of *J*_SC_ and *V*_OC_ on light intensity (P) to clarify the bimolecular recombination behavior of the PM6:L8-BO system. When the slope of *J*_SC_ versus light intensity approaches 1, the probability of bimolecular recombination is minimized. On the other hand, if the slope of *V*_OC_ versus light intensity is close to kT/q, bimolecular recombination dominates [51]. A significant deviation from this value indicates enhanced trap-assisted recombination. As shown in Figure 4a, the slopes for pure 2PACz and 2PACz + 2ICzMPE are 0.934 and 0.944, respectively, indicating that incorporation of the dispersants further suppresses bimolecular recombination. Meanwhile, Figure 4b shows that the slopes of *V*_OC_ versus light intensity are 1.030 and 1.015 kT/q, respectively, indicating that bimolecular recombination dominates in both devices. [52]. Furthermore, the charge separation and collection behavior were analyzed based on the relationship between the photocurrent density (*J*_ph_) and effective voltage (*V*_eff_) (Figure S10). At a high effective voltage (exceeds 2V), *J*_ph_ approaches saturation, corresponding to the saturated photocurrent density (*J*_sat_). Based on this, the charge dissociation probability *P*_diss_ can be calculated using the ratio *J*_sc_/*J*_sat_. The results show that the charge dissociation probability (*P*_diss_) increases from 98.1% in the control device to 99.0% upon the introduction of 2ICzMPE. A higher ratio indicates a stronger charge extraction capability, which is more favorable for achieving higher *J*_SC_ and FF. Moreover, this improvement aligns well with the previously observed suppression of bimolecular recombination, further confirming that interface engineering optimizes both charge generation and extraction processes [53]. In addition, the hole mobility (*μ*_h_) was further characterized using the space charge limited current (SCLC) method. As shown in Figure 4c, the introduction of 2ICzMPE, the *μ*_h_ increased from 7.01 × 10^−4^ cm^2^ V^−1^ s^−1^ to 7.70 × 10^−4^ cm^2^ V^−1^ s^−1^, indicating that the dispersant-regulated interface facilitates hole injection and transport, thereby enhancing charge transport capability [54,55,56,57].

To further correlate the enhanced transport distance of charge-separation (CS) with the suppression of photogenerated charge recombination, the drift length (*L*_Dr,CS_) and diffusion length (*L*_Dif,CS_) of CS states were quantitatively analyzed (Figure 4d,e) [58]. The *L*_Dr,CS_ can be expressed in terms of the figure of merit (FOM) *θ*, which characterizes the competition between the recombination rate and extraction rate:

$$L_{Dr,CS}=\sqrt{\frac{x_{0}^{2}}{\theta }}=\sqrt{\frac{q\mu_{n}\mu_{p}V_{int}}{\gamma x_{0}J_{sat}}}$$

where *q* is the elementary charge, Vint is the built-in voltage, *μ*_n_ and *μ*_p_ are electron (Figure S11) and hole mobilities, γ is the bimolecular recombination coefficient, and *J*_sat_ is the saturated photocurrent measured under a sufficiently large reverse bias (Table S5). Based on these measurements, the FOM *θ* decreases from 4.6 × 10^−3^ to 3.8 × 10^−3^ upon introducing 2ICzMPE. This reduction corresponds to an increase in the drift length of *L*_Dr,CS_ from 1247 nm to 1381 nm, indicating that carriers can travel longer distances under the built-in field before recombination.

The *L*_Dif,CS_ is expressed as follows [59]:

(1) $$L_{Dif,CS}=\sqrt{\frac{x_{0}^{2}}{2\alpha }}={\left(\frac{\mu_{n}\mu_{p}{\left(k_{B}T\right)}^{2}x_{0}}{q\gamma J_{sat}}\right)}^{\frac{1}{4}}$$

where α reflects the balance between photogenerated charge recombination and extraction in transport-limited solar cells, *k*_B_ is the Boltzmann constant, and *T* is the temperature. Upon introducing 2ICzMPE, *L*_Dif,CS_ increases from 53 nm to 58 nm, indicating enhanced carrier diffusion capability. The simultaneous increase in both the *L*_Dr,CS_ and *L*_Dif,CS_ demonstrates that carrier transport is optimized under both field-driven and concentration-gradient-driven conditions, allowing more efficient charge collection before recombination [60]. This enhancement directly reduces parallel resistance losses and suppresses space-charge accumulation, leading to a marked improvement in the FF. Experimentally, the FF increases from 79% to 81%, which is consistent with the observed trends in charge transport lengths. We attribute this improvement primarily to the dispersant-mediated regulation of the SAM interface: a more uniform interface reduces the trap-state density and suppresses recombination, while dipole-induced interface modulation strengthens the built-in field, facilitating rapid carrier extraction. The combined effect of these factors results in efficient charge transport, thereby significantly boosting the FF.

We analyzed the energy losses (Figure 4f) to see what caused the change in *V*_OC_. The photovoltaic bandgap, Shockley–Queisser (SQ) limit voltage, radiative limit, and electroluminescence external quantum efficiency (EQE_EL_) were extracted, allowing the three energy loss components to be summarized in Table S6 [60]. The introduction of 2ICzMPE leaves the optical bandgap unchanged (*E*_g_ = 1.41 eV, Figure S12a) but results in a higher q*V*_OC_ (0.859 eV vs. 0.867 eV) relative to the control device. A statistical analysis of energy losses (Figure 4f, Table S6) reveals that Δ*E*_1_ remains unchanged at 0.263 eV, whereas Δ*E*_2_ shows a negligible change. Non-radiative recombination losses were further evaluated through electroluminescence quantum efficiency measurements (EQE_EL_) (Figure S12b). The significantly higher EQE_EL_ observed after introducing 2ICzMPE directly evidences the suppression of non-radiative recombination pathways, showing non-radiative energy loss (Δ*E*_3_) decreasing from 0.203 eV to 0.196 eV, which is consistent with our energy loss decomposition. The enhanced EQE_EL_ implies that the 2ICzMPE interface reduces trap-assisted recombination, likely due to more uniform SAM coverage and better energy-level alignment. These results indicate that 2ICzMPE effectively suppresses carrier recombination losses, thereby improving *V*_OC_ [61].

Additionally, the device reliability was further evaluated through storage and light stability tests (Figure 4g,h) using unencapsulated devices. In a nitrogen atmosphere (25 °C, open-circuit), compared with 85.5% for the control device, the device maintained 90.8% of its initial PCE after 1500 h. Under continuous illumination (0.26mW cm^−2^, 25 °C, LED, open-circuit), the 2ICzMPE-modified device retained 86.6% of its initial PCE after 1500 h, versus 79.9% for the control device, demonstrating excellent photostability even without encapsulation. These results are noteworthy given that the devices were not encapsulated, highlighting the intrinsic stability imparted by the 2ICzMPE interface [62,63]. Collectively, the data indicate that the 2ICzMPE strategy is effective for laboratory-scale small-area devices and offers promise for practical applications.

## 3. Materials and Methods

Materials: 3,6-Diiodocarbazole,1-bromo-2-(2-methoxyethoxy)ethane, sodium hydride (NaH), N,N-dimethylformamide (DMF), ethyl acetate, n-hexane, silica gel, and ethanol were purchased from Hangzhou Aode Technology Co., Ltd. (Hangzhou, China). PM6 and L8-BO were purchased from Solarmer Materials Inc. (Los Angeles, CA, USA). TCB(1,3,5-Trichlorobenzene) was purchased from Tokyo Chemical Industry (TCI, Tokyo, Japan). PNDIT-F3N was purchased from eFlexPV Inc. (Los Angeles, CA, USA). 2PACz was purchased from Suzhou Liwei New Material Technology Co., Ltd. (Suzhou, China). CF, methanol, and ethanol were purchased from Sigma-Aldrich (St. Louis, MO, USA).

Methods: 3,6-Diiodo-9H-carbazole (500 mg, 1.13 mmol) and Sodium hydride (28.64 mg, 2.26 mmol) were placed in a flame-dried Schlenk flask under argon protection and dissolved in anhydrous N,N-dimethylformamide (DMF, 10 mL). The reaction mixture was stirred at room temperature for 30 min. 1-Bromo-2-(2-methoxyethoxy) ethane (436.85 mg, 2.26 mmol) was subsequently introduced via syringe, followed by heating at 70 °C overnight under continuous argon atmosphere. After completion of the reaction (monitored via thin-layer chromatography), the mixture was cooled to room temperature and carefully quenched with water. The resulting solution was extracted with ethyl acetate (3 × 20 mL), and the combined organic layers were washed successively with water and brine and then dried over anhydrous sodium sulfate. After filtration, the solvent was removed under reduced pressure to afford the crude product. The crude residue was purified via column chromatography on a silica gel using hexane/ethyl acetate (5:1, *v*/*v*) as the eluent to afford the desired product as a white solid (410 mg, 66% yield).

The BHJ devices were fabricated in a traditional device structure of ITO/2PACz/active layer/PNDIT-F3N/Ag. Patterned ITO substrates were first cleaned via successive ultrasonication in glass-cleaning solution, deionized water, acetone, and isopropanol. Prior to the deposition of the 2PACz layer, the substrates were subjected to ultraviolet ozone treatment for 20 min, followed by plasma cleaning for 5 min. For the control devices, the 2PACz solution (0.2 mg/mL in ethanol) was spin-coated at 3000 rpm for 30 s and then thermally annealed at 100 °C for 10 min. For the modified devices, 2ICzMPE was added into the 2PACz solution at a weight ratio of 1:10 (2ICzMPE:2PACz), followed by spin-coating under the same conditions and subsequent thermal annealing at 100 °C for 10 min. Then, PM6:L8-BO blend solutions were prepared in CF, with a fixed acceptor concentration of 7.3 mg/mL for PM6 and 8.7 mg/mL for L8-BO, respectively. The additive (TCB) 10 mg/mL was introduced during solution preparation. After stirring on 80 °C hotplate for over 2 h, the blend solutions were spin-coated onto the 2PACz layer to form the active layers. The blend solution was spin-coated at 2500 rpm for 30 s. Subsequently, a PNDIT-F3N layer was deposited via spin-coating (0.5 mg/mL in methanol with 0.5 vol% acetic acid, 2000 rpm, 30 s) onto the active layer. Finally, 100 nm Ag was deposited at a vacuum level of <1 × 10^−4^ Pa. The typical device area (0.0216 cm^2^) was defined based on a metal mask. The *J*-*V* characteristics were measured under simulated AM 1.5G illumination (100 mW cm^−2^) using a forward scan from −0.2 V to 1.0 V with a voltage step of 0.02 V and a delay time of 10 ms.

## 4. Conclusions

In summary, we have developed a dipolar dispersant molecule, 2ICzMPE, to address the aggregation of SAM molecules on ITO substrates based OSCs, which leads to non-uniform coverage, local defects, and severe interfacial charge recombination. As a proof of concept, we introduced 2ICzMPE into the 2PACz, then fabricated PM6:L8-BO OSCs, improving the PCE from 17.51% to 18.67%. By introducing 2ICzMPE as a dispersant, we effectively suppress the self-aggregation of the 2PACz through intermolecular push–pull interactions. Consequently, 2ICzMPE promotes the formation of a uniform SAM, as evidenced by AFM and surface potential mapping. These interfacial benefits directly translate into enhanced device performance in small-area OSCs. Through carrier dynamics characterization and simulation, the key role of the dispersant in achieving a higher dipole moment is confirmed, which greatly shortens the hole transport time, thereby facilitating efficient charge extraction and optimizing film morphology. Moreover, unencapsulated small-area devices exhibit excellent stability, retaining 86.6% of the initial PCE after 1500 h under continuous illumination and 90.8% after 1500 h of nitrogen storage. Collectively, our results demonstrate that 2ICzMPE is an effective interfacial regulator that simultaneously boosts efficiency and stability, offering a practical strategy for SAM-based interface engineering in organic photovoltaics.

## Data Availability

The original contributions presented in this study are included in the article. Further inquiries can be directed to the corresponding author.

## Supplementary Materials

The following supporting information can be downloaded at: https://www.mdpi.com/article/10.3390/molecules31132321/s1, Figure S1. ^1^H NMR spectrum of compound 2ICzMPE. Figure S2. ^13^C NMR spectrum of compound 2ICzMPE. Figure S3. HRMS spectrum of compound 2ICzMPE. Figure S4. The TGA curves of 2ICzMPE. Figure S5. Water contact angle of ITO substrates. Figure S6. Average fiber width of 2PACz SAMs before (a) and after (b) the incorporation of 2ICzMPE, determined from line-scan analysis of AFM height images. Figure S7. (a) (b) XPS spectra of P 2p, In 3d under different interfacial modification conditions. Figure S8. (a) *J*-*V* characteristics of PM6:L8-BO-based devices with 2PACz and 2PACz/2ICzMPE at different doping concentrations; (b) EQE spectra of PM6:L8-BO-based devices with 2PACz and 2PACz/2ICzMPE. Figure S9. Line profiles were drawn along the diagonal direction to determine the average fibril width of PM6:L8-BO blend films deposited on 2PACz and 2PACz/2ICzMPE-modified substrates. Figure S10. *J*_ph_-*V*_eff_ characteristics of devices based on 2PACz and 2PACz/2ICzMPE. Figure S11. Electron mobility of the device. Figure S12. (a) UV-vis absorption spectra of active layer films based on 2PACz and 2PACz/2ICzMPE. EQE_EL_ of devices based on 2PACz and 2PACz/2ICzMPE; (b) EQE_EL_ of devices based on 2PACz and 2PACz/2ICzMPE. Table S1. Photovoltaic properties of PM6:L8-BO devices upon adding different ratios of 2ICzMPE. Table S2. Statistics of photovoltaic parameters (*V*_OC_, *J*_SC_, FF, PCE). Table S3. Statistical photovoltaic parameters (*V*_OC_, *J*_SC_, FF, and PCE) of PM6:L8-BO devices based on Me-4PACz and 4PADCB SAMs. Table S4. Fitting parameters of GIWAXS. Table S5. Drift-diffusion simulation parameters. Table S6. Detailed energy loss analysis of devices based on 2PACz and 2PACz/2ICzMPE. References [64,65,66,67,68] are cited in the Supplementary Materials.
