# Supplementary material for: A Flexible Side-Chain Dispersant Enables Uniform Self-Assembled Monolayers for 18.67% Organic Solar Cells
Source: Molecules. 2026 Jul 2;31(13):2321. doi: 10.3390/molecules31132321 (PMC13363529; doi:10.3390/molecules31132321)
Supplement: Supplementary file 1 [file molecules-31-02321-s001.zip › molecules-4385636-supplementary.pdf]

Supporting Information

A Flexible Side-Chain Dispersant Enables Uniform Self-Assembled Monolayers for 18.67% Organic Solar Cells

Mengmeng Wang [1,2], Shibo Wang [2], Yabing Tang [2], Yuyan Li [2], Mengyu Qiu [2], Heng Liu [3], Leying Zha [2], Yajing Zhang [2,], Xinhui Lu [3] and Guilong Cai [1,2,4,*]*

[1]*School of Chemical and Molecular Sciences, Henan University (Zhengzhou), Zhengzhou 450000, China*

[2]*Longzihu New Energy Laboratory, Zhengzhou 450000, China*

[3]*Department of Physics, The Chinese University of Hong Kong, New Territories , Hong Kong 999077, China*

[4]*Beijing Key Laboratory of Solid State Battery and Energy Storage Process, Institute of Process Engineering Chinese Academy of Sciences, Beijing 100190, China*

**Corresponding author. yjzhang@ipezz.ac.cn (Y.Z.); glcai@ipe.ac.cn (G.C.)*

Experimental Instrumentation

The *J-V* curves were performed in the N_2-filled glovebox under AM 1.5G (100 mW cm^{-2}) using an AAA solar simulator (SS-X50, Enlitech) and a programmable sourcemeter (B2901BL, Enlitech), calibrated by the certified standard silicon solar cell (SRC-2020, Enlitech). The EQE curves were recorded through the solar cell spectral response measurement system (QE-R, Enlitech) with calibrated light intensity by a standard Si photovoltaic cell. Transient Photovoltage (TPV) and Transient photocurrent (TPC) was detected by an integrated testing system (TPCV, Enlitech). The EQE_{EL} measurement was performed on REPS-PRO (Enlitech). The absorbance was obtained on a Shimadzu UV-3600 Plus Spectrophotometer. The TM-AFM was measured by Cypher ES (Oxford Instruments). Contact angle measurement was performed on Biolin Theta Lite. The temperature was controlled by the OXFORD attachment. GIWAXS was conducted on active layer films at the Brockhouse X-ray Diffraction and Scattering Sector Low Energy Wiggler (BXDS-WLE) beamline of the Chinese University of Hong Kong using a photon energy of 15.12 keV (λ = 0.82 Å). The XPS and UPS measurements were carried out using a Shimadzu AXIS SUPRA+ X-ray photoelectron spectrometer. The binding energy scale of the XPS spectra was calibrated with reference to the adventitious carbon C 1s peak at 284.8

eV. The work function was determined from the secondary electron cutoff of the UPS spectra. The ^{1}H NMR and ^{13}C NMR spectra were recorded on a Bruker AVANCE NEO 600 MHz liquid nuclear magnetic resonance spectrometer. High-resolution mass spectrometry (HRMS) measurements were performed on an AB Sciex TripleTOF 5600+ system.

Calculating the SCLC mobility [64,65]

The SCLC mobility (μ) was measured with the hole-only device structure of ITO/2PACz or 2PACz-2ICzMPE/active layer/MoO_3/Ag and electron-only device structure of ITO/ZnO/active layer/PNDIT-F3N/Ag. ZnO was deposited by spin-coating a ZnO precursor solution (zinc acetate dihydrate, dissolved in 2-methoxyethanol with ethanolamine) on the ITO substrates at a speed of 4500 rpm for 30 s, followed by thermal annealing at 200 °C for 30 min. The values of SCLC mobility were obtained by fitting the current density-voltage curves according to

$$J = \frac{9\varepsilon_0 \varepsilon_r \mu V^2}{8L^3} \quad \text{(S1)}$$

Here, ε_0 is the vacuum dielectric constant (8.85×10^{-12} F^{-1} m^{-1}), ε_r is the relative dielectric constant (assumed to be 3 here), and L is the film thickness.

Calculating the energy loss [66–68]

Based on detailed balance and reciprocity, energy loss can be expressed as the sum of three components, as shown below:

$$\begin{aligned} q\Delta V_{\text{loss}} &= \Delta E_1 + \Delta E_2 + \Delta E_3 \\ &= \left(E_{\text{g}} - qV_{\text{OC}}^{\text{SQ}}\right) + \left(qV_{\text{OC}}^{\text{SQ}} - qV_{\text{OC}}^{\text{rad}}\right) + \left(qV_{\text{OC}}^{\text{rad}} - qV_{\text{OC}}\right) \\ &= \left(E_{\text{g}} - qV_{\text{OC}}^{\text{SQ}}\right) + qV_{\text{OC}}^{\text{rad,below gap}} + qV_{\text{OC}}^{\text{nrad}} \end{aligned} \quad \text{(S2)}$$

where ΔV_{loss} is the total voltage loss, E_{g} is band gap, $V_{\text{OC}}^{\text{SQ}}$ is the maximum voltage deduced by the Shockley-Queisser, $V_{\text{OC}}^{\text{rad}}$ is the open-circuit voltage when there is only radiative recombination, $\Delta V_{\text{OC}}^{\text{rad, below gap}}$ is the voltage loss of radiative recombination from the absorption below the bandgap, $\Delta V_{\text{OC}}^{\text{nrad}}$ is the voltage loss of non-radiative recombination.

The intersect of the extrapolated baseline and absorption edge defines the $\lambda_{\text{on-set}}$, and then the E_{g} can be calculated by $E_{\text{g}} = 1240/\lambda_{\text{on-set}}$.

In order to calculate the energy loss parameters, we should firstly understand where the

losses are from. The V_{OC} of any type of solar cells is determined by the ratio between short circuit current (J_{SC}) and dark saturation current (J_0), following this expression:

$$V_{oc} = \frac{k_B T}{q} \ln(\frac{J_{SC}}{J_0} + 1) \tag{S3}$$

where k_B is the boltzmann constant, T is the temperature, and q is the elementary charge. The expression for J_{SC} and J_0 are given by:

$$J_{SC} = q \cdot \int_0^{\infty} \mathrm{EQE_{pv}}(E) \cdot \varphi_{\mathrm{AM1.5}}(E) dE \tag{S4}$$

$$J_0 = \frac{q}{EQE_{\mathrm{EL}}} \cdot \int_0^{\infty} \mathrm{EQE_{pv}}(E) \cdot \varphi_{\mathrm{bb}}(E) dE \tag{S5}$$

The expression for J_0 is the Rau's reciprocity relation, where EQE_{EL} is radiative quantum efficiency of the solar cell when charge carriers are injected into the device in dark, $\varphi_{AM1.5}$ is the AM1.5 standard solar spectrum and φ_{bb} is the black body spectrum. When all the recombination is radiative (i.e. $EQE_{EL} = 1$), J_0 is minimized, and V_{OC} is maximized:

$$J_0^{rad} = q \cdot \int_0^{\infty} \mathrm{EQE_{pv}}(E) \cdot \varphi_{\mathrm{bb}}(E) dE \tag{S6}$$

$$V_{\mathrm{OC}}^{\mathrm{rad}} = \frac{k_B T}{q} \ln(\frac{J_{SC}}{J_0^{rad}} + 1) = \frac{k_B T}{q} \ln(\frac{q \cdot \int_0^{\infty} \mathrm{EQE_{pv}}(E) \cdot \varphi_{\mathrm{AM1.5}}(E) dE}{q \cdot \int_0^{\infty} \mathrm{EQE_{pv}}(E) \cdot \varphi_{\mathrm{bb}}(E) dE} + 1) \tag{S7}$$

In the Shockley-Queisser theory, the general quantum efficiency $EQE_{pv}^{SQ}(E)$ can be defined as follow:

$$\begin{cases} \mathrm{EQE_{PV}^{SQ}}(E) = 1, E > E_g \\ \mathrm{EQE_{PV}^{SQ}}(E) = 0, E < E_g \end{cases} \tag{S8}$$

Substituting general quantum efficiency $EQE_{pv}^{SQ}(E)$ (equation S16) in equations S12 and S14, then we can get the short circuit current and dark saturation current in the SQ limit:

$$J_{SC}^{SQ} = q \cdot \int_{E_g}^{\infty} \varphi_{\mathrm{AM1.5}}(E) dE \tag{S9}$$

$$J_0^{SQ} = q \cdot \int_{E_g}^{\infty} \varphi_{\mathrm{bb}}(E) dE \tag{S10}$$

In the same way, we can calculate the value of the SQ open-circuit voltage limit, V_{OC}^{SQ} according to equation S7:

$$V_{\mathrm{OC}}^{\mathrm{SQ}} = \frac{k_B T}{q} \ln(\frac{J_{SC}^{SQ}}{J_0^{SQ}} + 1) = \frac{k_B T}{q} \ln(\frac{q \cdot \int_{E_g}^{\infty} \varphi_{\mathrm{AM1.5}}(E) dE}{q \cdot \int_{E_g}^{\infty} \varphi_{bb}(E) dE} + 1) \tag{S11}$$

The difference between V_{OC}^{SQ} and V_{OC}^{rad} is due to that in the SQ theory, the band edge of the absorber is totally abrupt when calculating V_{OC}^{rad}, the band gap will be smeared out for the

existence of charge transfer state absorption.

Therefore, we can deduce the voltage loss of radiative recombination below the gap

$$V_{\mathrm{OC}}^{\mathrm{rad,below\ gap}} = V_{\mathrm{OC}}^{\mathrm{SQ}} - V_{\mathrm{OC}}^{\mathrm{rad}} \quad (S12)$$

The voltage loss due to non-radiative recombination, $V_{\mathrm{OC}}^{\mathrm{rad}}$, can be rewritten as

$$V_{\mathrm{OC}}^{\mathrm{nrad}} = V_{\mathrm{OC}}^{\mathrm{rad}} - V_{\mathrm{OC}} = -k_{\mathrm{B}}T \ln \mathrm{EQE}_{\mathrm{EL}} \quad (S13)$$

From which we can calculate the Voc value. Based on the previous discussions, we are now able to summarize the energy loss from the E_g to the qV_{OC} for any type of solar cells. We can get these three terms of energy losses based on related experiments and calculations.

Characterization of the compound

3,6-Diiodo-9-(2-(2-methoxyethoxy) ethyl)-9H-carbazole: ^{1}H NMR (600 MHz, acetone-d$_6$): δ 8.53 (t, J = 1.6 Hz, 2H), 7.74 (dd, J = 8.6, 1.6 Hz, 2H), 7.49 (dd, J = 8.6, 2.0 Hz, 2H), 4.56 (s, 2H), 3.87 (t, J = 5.4 Hz, 2H), 3.47 (dd, J = 5.5, 4.1 Hz, 2H), 3.34–3.30 (m, 2H), 3.14 (s, 3H).**^{13}C NMR (151 MHz，acetone-d$_6$):** δ 134.41，129.24，112.18，81.35，71.74，70.41，69.35，43.33. HRMS (ESI) m/z [M+H]$^+$, calcd for $C_{17}H_{17}I_2NO_2^+$:521.9382, found:521.9350.

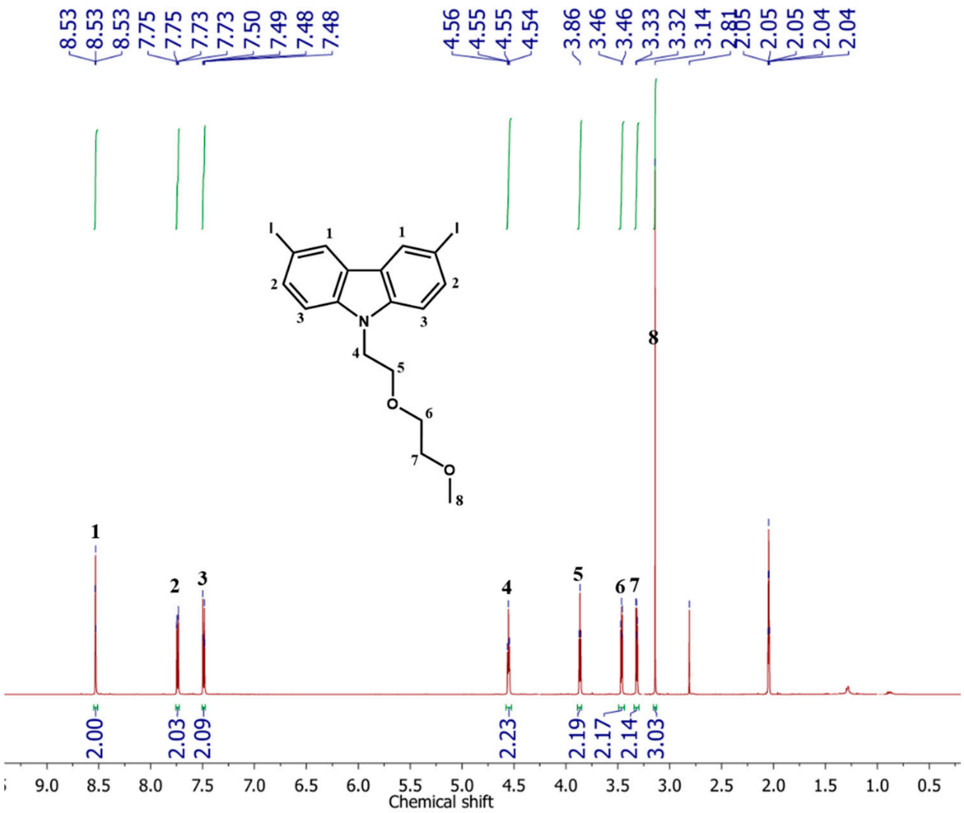

Figure S1. ^{1}H NMR spectrum of compound 2ICzMPE.

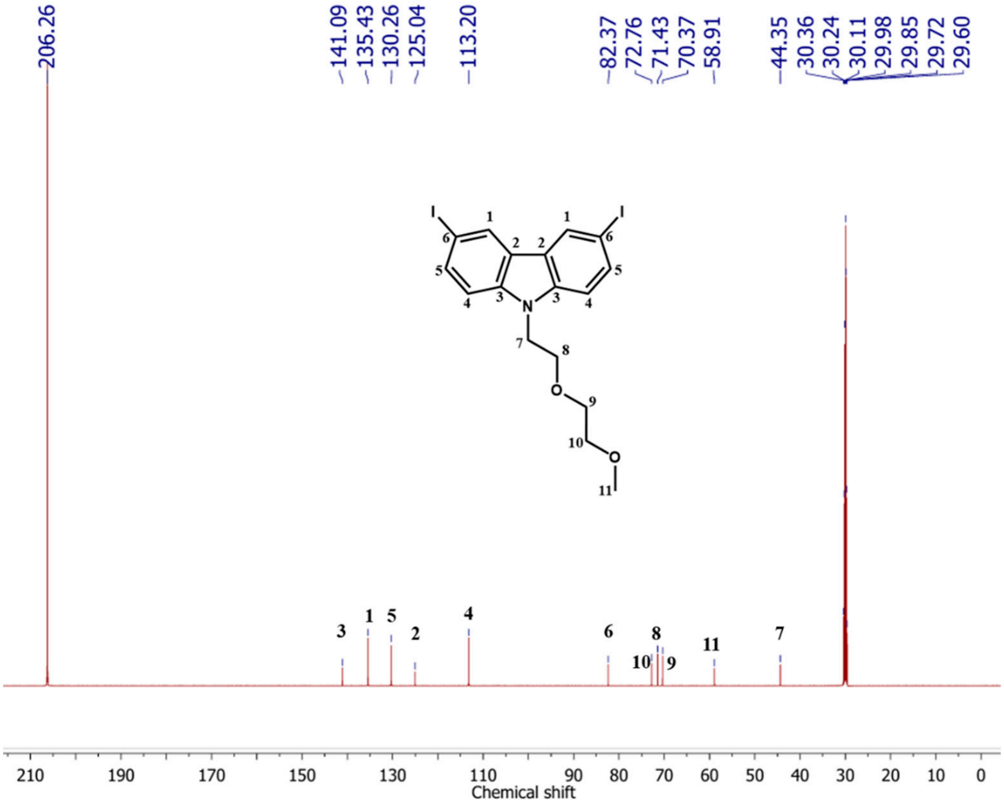

Figure S2. ^{13}C NMR spectrum of compound 2ICzMPE.

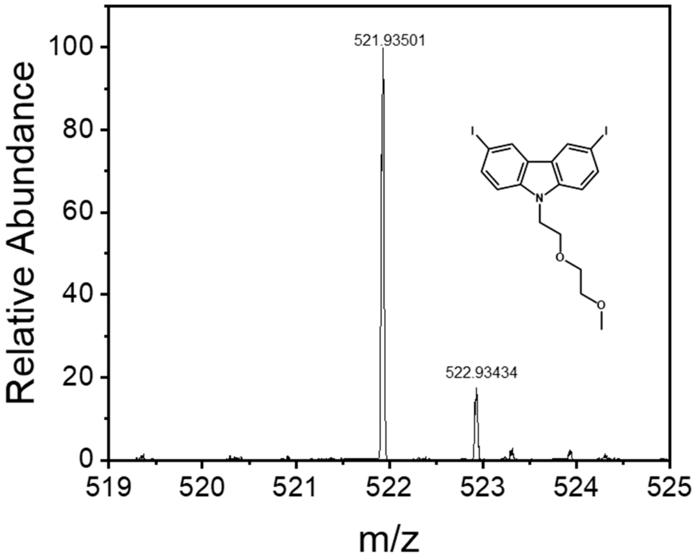

Figure S3. HRMS spectrum of compound 2ICzMPE.

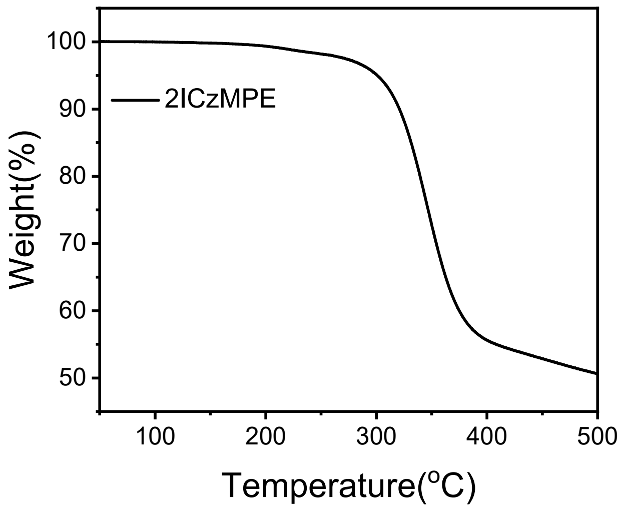

Figure S4. The TGA curves of 2ICzMPE.

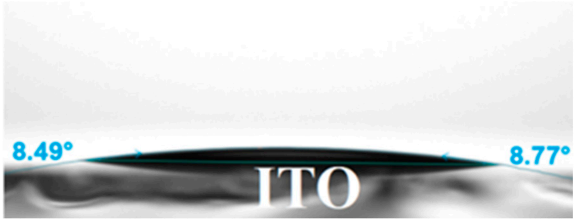

Figure S5. Water contact angle of ITO substrates.

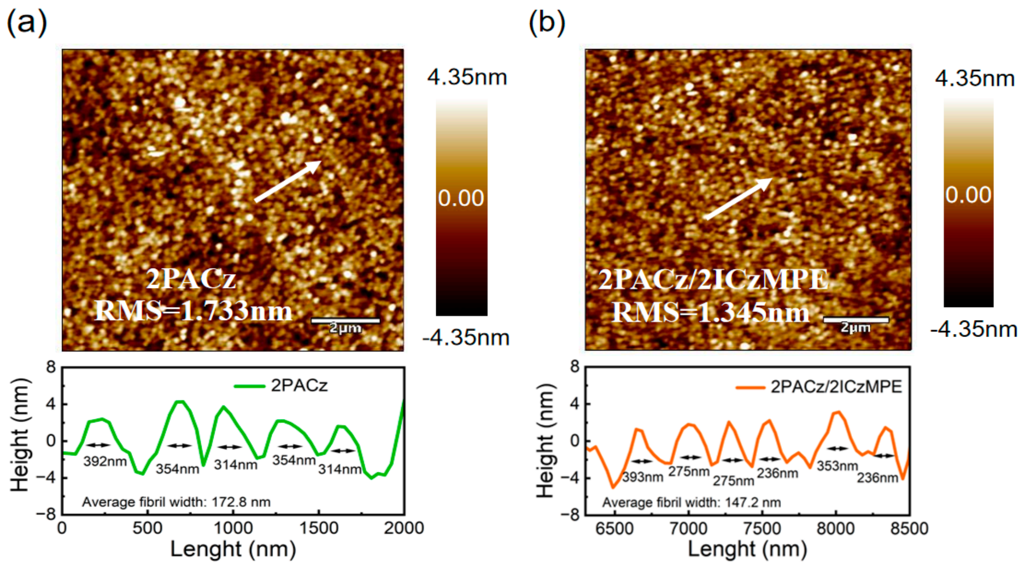

Figure S6. Average fiber width of 2PACz SAMs before (a) and after (b) the incorporation of 2ICzMPE, determined from line-scan analysis of AFM height images.

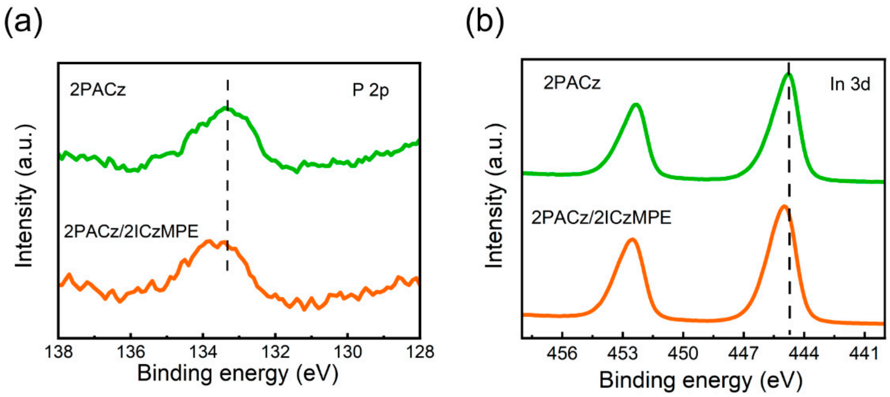

Figure S7. (a) (b) XPS spectra of P 2p, In 3d under different interfacial modification conditions.

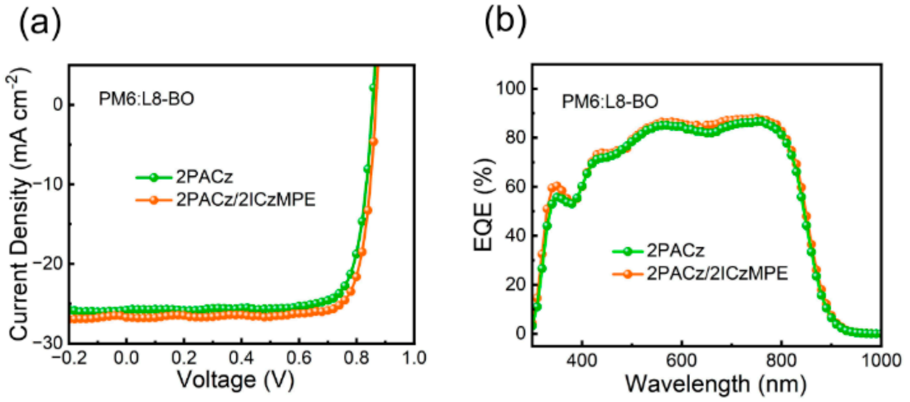

Figure S8. (a) *J–V* characteristics of PM6:L8-BO-based devices with 2PACz and 2PACz/2ICzMPE at different doping concentrations; (b) EQE spectra of PM6:L8-BO-based devices with 2PACz and 2PACz/2ICzMPE.

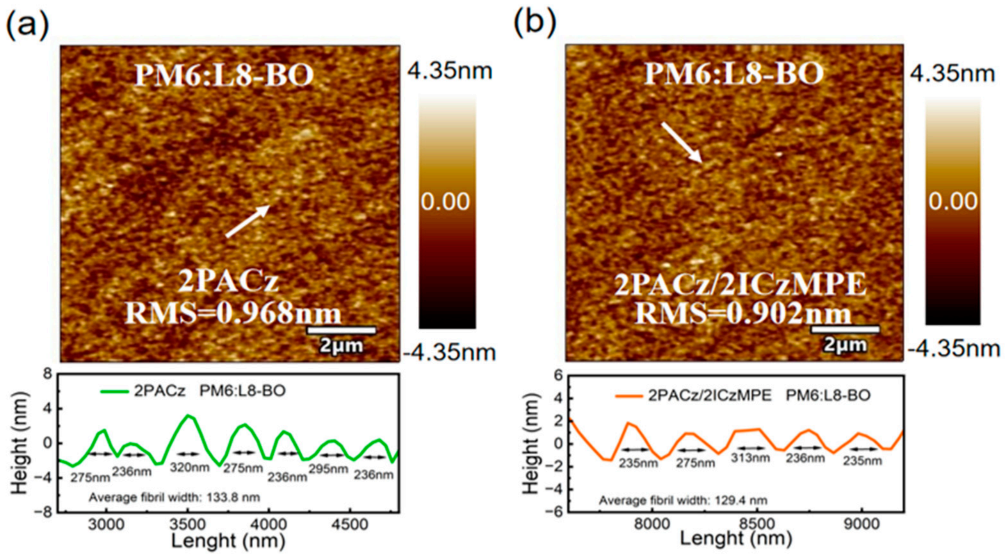

Figure S9. Line profiles were drawn along the diagonal direction to determine the average fibril width of PM6:L8-BO blend films deposited on 2PACz and 2PACz/2ICzMPE-modified substrates.

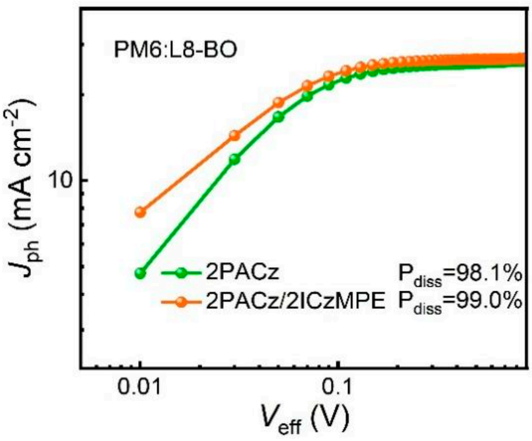

Figure S10. J_{ph}-V_{eff} characteristics of devices based on 2PACz and 2PACz/2ICzMPE.

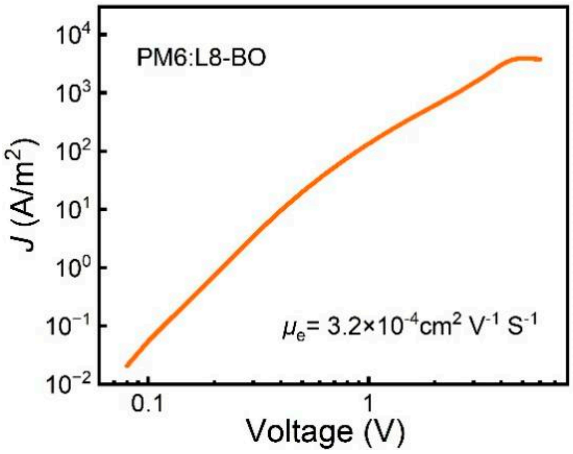

Figure S11. Electron mobility of the device.

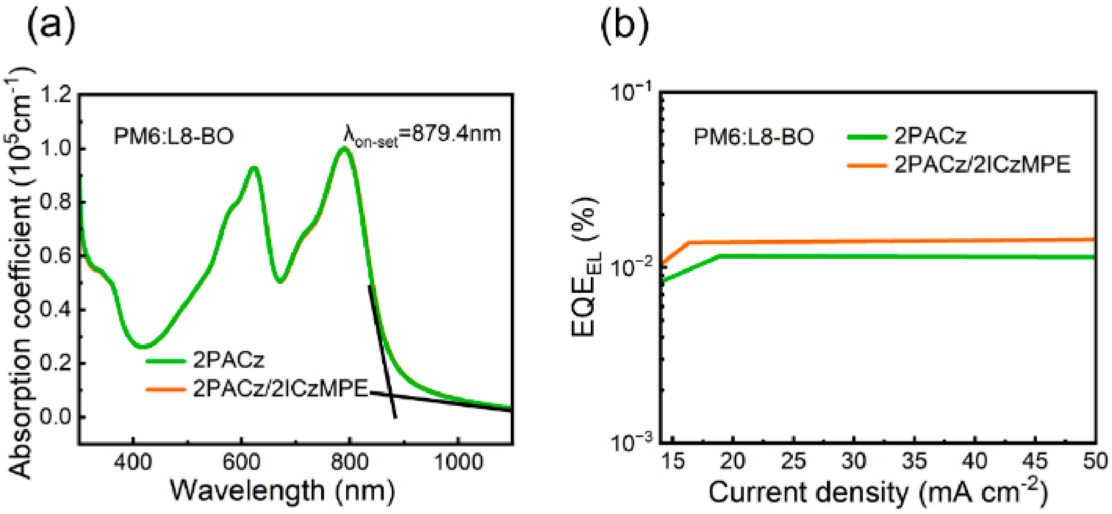

Figure S12. (a) UV-vis absorption spectra of active layer films based on 2PACz and 2PACz/2ICzMPE. EQE_{EL} of devices based on 2PACz and 2PACz/2ICzMPE; (b) EQE_{EL} of devices based on 2PACz and 2PACz/2ICzMPE.

Table S1. Photovoltaic properties of PM6:L8-BO devices upon adding different ratios of 2ICzMPE.

Conditions	V_{OC} (V)	J_{SC} (mA cm^{-2})	FF (%)	PCE (%)
PM6:L8-BO (2PACz:2ICzMPE) (8：1)	0.870 (0.866±0.003)	26.54 (26.37±0.13)	79.18 (79.44±0.20)	18.28 (18.14±0.10)
PM6:L8-BO (2PACz:2ICzMPE) (9：1)	0.866 (0.867±0.002)	26.63 (26.57±0.06)	80.19 (79.71±0.41)	18.48 (18.36±0.09)
PM6:L8-BO (2PACz:2ICzMPE) (10：1)	0.867 (0.868±0.001)	26.71 (26.49±0.14)	80.62 (80.11±0.48)	18.67 (18.44±0.15)
PM6:L8-BO (2PACz:2ICzMPE) (11：1)	0.867 (0.866±0.001)	26.79 (26.56±0.20)	79.90 (79.98±0.24)	18.56 (18.39±0.13)
PM6:L8-BO (2PACz:2ICzMPE) (12：1)	0.868 (0.868±0.003)	26.53 (26.37±0.13)	79.77 (79.99±0.18)	18.37 (18.31±0.08)

Table S2. Statistics of photovoltaic parameters (V_{OC}, J_{SC}, FF, PCE).

Conditions	V_{OC} (V)	J_{SC} (mA cm^{-2})	FF (%)	PCE (%)	J_{EQE} (mA cm^{-2})
PM6:L8-BO (2PACz)	0.859 (0.854±0.003)	25.88 (26.09±0.36)	78.77 (77.77±1.45)	17.51 (17.33±0.20)	24.82
PM6:L8-BO (2PACz/2ICzMPE)	0.867 (0.868±0.001)	26.71 (26.49±0.14)	80.62 (80.11±0.48)	18.67 (18.44±0.15)	25.44

The average value with standard deviation were obtained from 10 devices

Table S3. Statistical photovoltaic parameters (V_{OC}, J_{SC}, FF, and PCE) of PM6:L8-BO devices based on Me-4PACz and 4PADCB SAMs.

Conditions	V_{OC} (V)	J_{SC} (mA cm^{-2})	FF (%)	PCE (%)
PM6:L8-BO (Me-4PACz)	0.865 (0.859±0.003)	26.05 (26.05±0.02)	77.07 (76.22±1.66)	17.37 (17.08±0.29)
PM6:L8-BO (Me-4PACz/2ICzMPE)	0.867 (0.866±0.001)	26.79 (26.69±0.07)	79.53 (79.26±0.23)	18.47 (18.42±0.05)
PM6:L8-BO (4PADCB)	0.858 (0.856±0.003)	25.88 (25.75±0.31)	78.65 (77.26±1.94)	17.46 (17.16±0.32)
PM6:L8-BO (4PADCB/2ICzMPE)	0.868 (0.868±0.002)	26.77 (26.37±0.22)	79.37 (79.43±0.29)	18.45 (18.19±0.17)

Table S4. Fitting parameters of GIWAXS.

Conditions	Location (Å^{-1})	d-spacing (Å)	FWHM (Å^{-1})	CL (Å)	Area (a.u.)
2PACz	1.772	3.550	0.330	1.71	11.65
With 2ICzMPE	1.772	3.550	0.285	1.98	10.15

Table S5. Drift-diffusion simulation parameters.

Conditions	μ_h (10^{-4}cm^{2}V^{-1}s^{-1})	μ_e (10^{-4}cm^{2}V^{-1}s^{-1})	*d (nm)*	J_{sat} *(mA cm^{-2})*	β
2PACz	7.0	3.2	85	24.7	0.935
With 2ICzMPE	7.7	3.2	85	25.5	0.944

Conditions	$V_{OC}(V)$	γ_{pre} (10^{-2})	FOM θ (10^{-3})	L_{dr} *(nm)*	FOM α	L_{diff} (nm)
2PACz	0.861	1.50	4.6	1247.2	1.13	53.1
With 2ICzMPE	0.872	1.25	3.8	1381.7	1.04	58.0

Table S6. Detailed energy loss analysis of devices based on 2PACz and 2PACz/2ICzMPE.

Conditions	E_g (eV)	qV_{OC}^{SQ} (eV)	qV_{OC}^{rad} (eV)	ΔE_1 (eV)	ΔE_2 (eV)	ΔE_3 (eV)	$qV_{OC,cal}$ (eV)	qV_{OC} (eV)
2PACz	1.41	1.147	1.062	0.263	0.085	0.203	0.859	0.859
With 2ICzMPE	1.41	1.147	1.065	0.263	0.082	0.196	0.869	0.867
